# Supplementary material for: Silver Nanoparticles with Liquid Crystalline Ligands Based on Lactic Acid Derivatives
Source: Nanomaterials (Basel). 2019 Jul 25;9(8):1066. doi: 10.3390/nano9081066 (PMC6723366; doi:10.3390/nano9081066)
Supplement: Supplementary file 1 [file nanomaterials-09-01066-s001.pdf]

nanomaterials

Supplementary Materials

Silver Nanoparticles with Liquid Crystalline Ligands based on Lactic Acid Derivatives

Tinkara Troha [1], Miroslav Kaspar [1], Vera Hamplova [1], Martin Cigl [1], Jaroslav Havlicek [1], Damian Pociecha [2] and Vladimira Novotna [1,*]

1 Institute of Physics of the Czech Academy of Sciences, Prague 18221, Czech Republic
2 Faculty of Chemistry, University of Warsaw, ul. Zwirki i Wigury 101, 02-089 Warsaw, Poland
* Correspondence: novotna@fzu.cz

Content

1. Synthesis

2. Nanoparticle Preparation

3. Example of NMR Spectra of Free Thiol and Nanoparticle System

4. Results

5. References

1. Synthesis

Chiral thiol ligands were synthesized by the synthetic routes depicted in **Scheme S1**. Starting phenone **1** was synthesized following a known procedure from literature [S1] and similarly the chiral intermediates **4**, **6**, and 8—see Refs. [S2], [S3], and [S4], respectively. Starting phenone **1** was alkylated using excess of 1,10-dibromodecane. The alkylated phenone **2** was treated with a solution of hypobromite to afford the carboxylic acid **3**. Synthesized carboxylic acid **3** was then esterified by appropriate chiral hydroxy-compound (**4**, **6**, or **8**) via DCC-coupling yielding bromo-esters **5**, **7**, resp. **8**. In the last step, the bromo-esters **5**, **7**, and **8** were refluxed with sodium thiosulfate to obtain appropriate Bunte salt from which the target thiols **A–C** were formed under acidic conditions and under inert atmosphere of argon to prevent oxidation of thiol group.

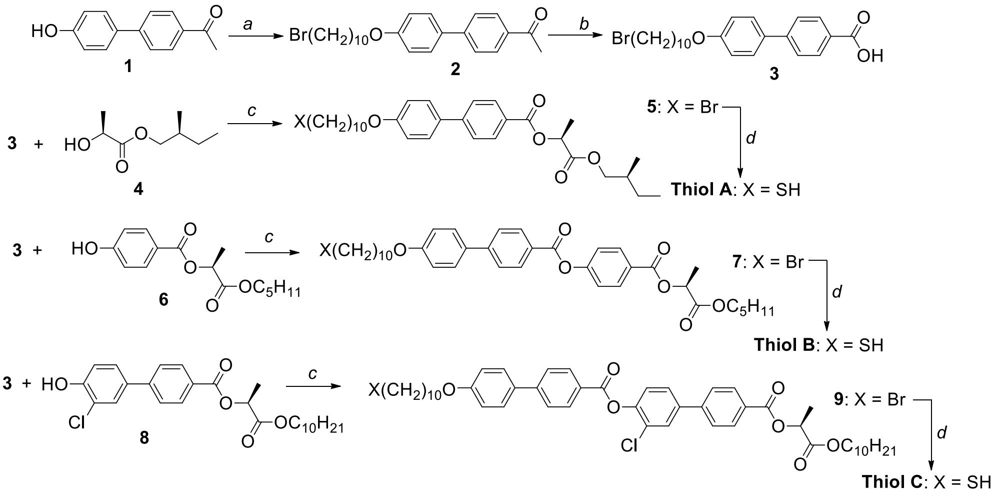

Scheme S1. Synthesis of thiol ligands **A–C**. Reagents and conditions: (**a**) 1,10-dibromodecane, KOH, dioxane-water, reflux 8 h; (**b**) 1. NaBrO, dioxane; 2. HCl; (**c**) DCC, DMAP, THF-CH_2Cl_2; (**d**) 1. $Na_2S_2O_3$, dioxane-EtOH-H_2O, reflux 12–40 h; 2. HCl, THF, Ar.

1.1. General Experimental

^{1}HNMR spectra were recorded on Varian VNMRS 300 instrument (Varian, Inc., Palo Alto, CA, USA). Deuteriochloroform ($CDCl_3$) and hexadeuteriodimethyl sulfoxide (DMSO-d_6) were used as solvents and signals of the solvent served as internal standard. Chemical shifts (δ) are given in ppm, and J values are given in Hz. The signals were identified by APT, gCOSY, and gHMBC experiments. The purity of the final compound was checked by HPLC analysis (high-pressure pump ECOM Alpha; column WATREX Biospher Si 100, 250 mm × 4 mm, 5 μm; detector WATREX UVD 250) and were found to be >99.8 %. Column chromatography was carried out using Merck Kieselgel 60 (60–100 μm). Enantiomeric purity of chiral compounds was confirmed by chiral HPLC system (chiral column: Daicel Chiralpak AD-3 (Chiral Technologies Europe SAS, Illkirch – Cedex, France), 150 mm× 4.6 mm I.D., 3 μm).

1.2. Synthetic Procedures

1. -(4'-((10-Bromodecyl)oxy)-[1,1'-biphenyl]-4-yl)ethanone (2)

A mixture of 1-(4'-hydroxy-[1,1'-biphenyl]-4-yl)ethanone (**1**) (42.4 g, 0.2 mol) 1,10-dibromodecane (120.0 g, 0.4 mol) and potassium hydroxide (12.3 g, 0.22 mol) in dioxane (400 mL)–water (40 mL) mixture was refluxed with intensive stirring for 8 h. When cooled to room temperature, the resulting mixture was poured into water (500 mL) and the precipitate filtered off, washed with water, and dried. Crude product was recrystallized from toluene (500 mL) yielding pure phenone **2** 54.3 g (63 %). ^{1}H NMR (DMSO-d_6): 8.08 (2 H, d, *J* = 8.2, H-3, H-5), 7.76 (2 H, d, *J* = 8.2, H-2, H-6), 7.69 (2 H, d, *J* = 8.5, H-2′, H-6′), 7.04 (2 H, d, *J* = 8.5, H-3′, H-5′), 4,0 (2 H, t, *J* = 6.5, OCH_2), 3.52 (t, 2 H, *J* = 6.7, CH_2Br), 2.58 s, 3 H, ($COCH_3$), 1.69–1.83 (m, 4 H, $\mathbf{CH_2}CH_2O$, $\mathbf{CH_2}CH_2Br$), 1.20–1.48 (m, 12 H $(CH_2)_6$).

4. '-((10-Bromododecyl)oxy)-[1,1'-biphenyl]-4-carboxylic acid (3)

Phenone **2** 52.0 g (0.11 mol) dissolved in dioxane (1.0 l) was slowly treated at 40 °C with a solution of sodium hypobromite prepared by mixing of bromine (20 mL, 0.39 mol) with solution of sodium hydroxide (300 mL, 20%) at 0 °C. After 4 h of stirring at 40 °C, the reaction mixture was allowed to cool to room temperature and the precipitate filtered off and washed with water (200 mL), acidified with hydrochloric acid (100 mL, 1:1), and washed with water again. After drying in vacuum drier, the crude product was boiled with toluene, and solid was filtered and further recrystallized from isopropanol. Yield 40.2 g (76 %). ^{1}H NMR (DMSO-d_6): 7.96 (2 H, d, *J* = 8.2, H-3, H-5), 7.73 (2 H, d, *J* = 8.2, H-2, H-6), 7.65 (2 H, d, *J* = 8.8, H-2′, H-6′), 7.01 (2 H, d, *J* = 8.8, H-3′, H-5′), 3,99 (2 H, t, *J* = 6.5, OCH_2), 3.54 (t, 2 H, *J* = 6.7, CH_2Br), 1.67–1.84 (m, 4 H, $\mathbf{CH_2}CH_2O$, $\mathbf{CH_2}CH_2Br$), 1.21–1.45 (m, 12 H $(CH_2)_6$).

(S,S)-1-(2-methylbutoxy)-1-oxopropan-2-yl 4'-((10-bromodecyl)oxy)-[1,1'-biphenyl]-4-carboxylate (5)

To a mixture of acid **3** (31.0 g, 71.53 mmol) and chiral phenol **4** (11.46 g, 72.40 mmol) in dichloromethane-THF mixture (150 mL + 150 mL) cooled to 2–8 °C was added *N,N′*-dicyclohexylcarbodiimide (DCC, 15.0 g, 72.70 mmol) and *N,N*-(dimethylamino)pyridine (2.2 g, 18.16 mmol). The reaction mixture was stirred for 6 h, during which the temperature was allowed to reach the room temperature. Precipitate was filtered off, and the filtrate evaporated under reduced pressure. The residue was purified by means of column chromatography on silica using dichloromethane-acetone (99:1) as eluent. Yield 18.70 g (45 %).^{1}H NMR ($CDCl_3$): 8.12 (2 H, d, H-3, H-5), 7.60 (4 H, m, H-2, H-6, H-2′, H-6′), 7.00 (2 H, d, H-3′, H-5′), 5.38 (1 H, q, *J* = 7.04, CH*), 3.93–4.11 (4 H, m, CH_2OAr, CH_2C^*), 3.40 (2 H, t, CH_2Br), 1.20–1.80 m (19 H, CH_2, CH), 0.84–0.92 m (6 H, 2 × CH_3).

(S,S)-1-(2-methylbutoxy)-1-oxopropan-2-yl 4'-((10-sulfanyldecyl)oxy)-[1,1'-biphenyl]-4-carboxylate (Thiol A)

Bromo-ester **5** (16.0 g, 27.80 mmol) was refluxed for 12 h in the solution of sodium thiosulphate (10.0 g, 63.25 mmol) in dioxane/water mixture (250 mL + 50) being catalysed by potassium iodide (1.0 g). After cooling to ambient temperature, the crude salt ("Bunte salt") was filtered off and crystallized twice from ethanol. Then, the product was dissolved under argon atmosphere in the mixture of tetrahydrofurane (50 mL) and of diluted hydrochloric acid (20 mL, 10 %) and stirred for one hour. After that, water (500 mL) and benzene (400 mL) were added, and the intensive shaking in argon atmosphere continued. The organic layer was washed twice with water and dried by magnesium sulphate. The solvent was evaporated, leaving white product **Thiol A** (5.8 g, 38 %). ^{1}H NMR of **4** (C_6D_6): 8.19 (2 H, d, H-3, H-5), 7.35 – 7.60 (4 H, m, H-2, H-6, H-2′, H-6′), 6.87 (2 H, d, H-3′, H-5′), 5.38 (1 H, q, *J* = 7.04, CH*), 3.90 (2 H, t, *J* = 6.5, OCH_2), 3.73 (t, 2 H, *J* = 6.7,$COOCH_2C$*), 2.20 (2 H, m, CH_2S); 1.00–1.70 (19 H, m, CH_2, CH); 0.73 (3 H, m, 2 × CH_3). *The signals of residual disulphide: 2.30 (0.03 H, m, CH_2-S-S-CH_2).*

(S)-4''-(((1-oxo-1-(pentyloxy)propan-2-yl)oxy)carbonyl)phenyl 4'-((10-bromodecyl)oxy)-[1,1'-biphenyl]-4-carboxylate (7)

Using a procedure described for **5**, acid **3** (16.0 g, 33.3 mmol) and chiral phenol **6** (10.5 g, 37.5 mmol) in dichloromethane-THF mixture (150 mL + 150 mL) were condensed in the presence of DCC (7.5 g, 36.4 mmol) and DMAP (1.1 g, 9.0 mmol). The crude product was purified by means of column chromatography on silica using dichloromethane-acetone (99.5:0.5) as eluent. Yield 21.0 g (85 %). ^{1}H NMR ($CDCl_3$): 8.18–8.26 (4 H, m, H-3, H-5, H-3′′, H-5′′), 7.71 (2 H, d, *J*=8.2, H-2, H-6), 7.61 (2 H, d, *J*=8.8, H-2′, H-6′), 7.35 (2 H, d, *J*=8.8, H-2′′, H-6′′), 7.02 (2 H, d, *J*=8.8, H-3′, H-5′), 5.35 (1 H, q, *J*=7.04, CH*), 4.10 – 4.28 (2 H, m, $COOCH_2$), 4.03 (2 H, t, *J*=6.7, OCH_2), 3.53 (2 H, t, *J* = 7,0, CH_2Br), 1.77–1.96 (4 H, m, C**H**$_2CH_2O$, C**H**$_2CH_2Br$), 1.58–1.72 (5 H, m, C**H**$_2CH_2OOC$, C**H**$_3CH$*), 1.21–1.50 (16 H, m, 8 × CH_2), 0.88 (t, 3 H, *J* = 6.7, CH_2C**H**$_3$).

(S)-4''-(((1-oxo-1-(pentyloxy)propan-2-yl)oxy)carbonyl)phenyl 4'-((10-sulfanyldecyl)oxy)-[1,1'-biphenyl]-4-carboxylate (Thiol B)

Bromo-ester **7** (10.0 g, 13.5 mmol) was dissolved in a mixture of dioxane and ethanol (150 mL + 150 mL), and a solution of sodium thiosulphate pentahydrate (20.0 g, 80.6 mmol) in water (30 mL) was added with stirring. Reaction mixture was refluxed for 12 h. The resulting cooled mixture was then placed overnight in a refrigerator (−25 °C). Crystallized solid was filtered off and washed with small amount of ethanol and water. Crude Bunte salt was recrystallized from the ethanol-dioxane mixture.

The solution of Bunte salt in THF (300 mL) was stirred with hydrochloric acid (100 mL, 1:2) for 30 min under argon atmosphere. Then, benzene was added (400 mL), and the mixture was transferred to separation funnel and shaken properly. Acetonitrile (100 mL) was added to the resulting turbid solution, and after clearing, the organic layer was separated and washed with water, still under argon atmosphere. The organic layer was dried over anhydrous magnesium sulphate, filtered, and the clear solution evaporated under inert atmosphere. Yield 4.5 g (51 %). ^{1}H NMR (C_6D_6, 60°C): 8.12–8.25 (4 H, m, H-3, H-5, H-3′′, H-5′′), 7.42 (2 H, d, *J* = 8.2, H-2, H-6), 7.38 (2 H, d, *J*=8.8, H-2′, H-6′), 7.01 (2 H, d, *J* = 8.8, H-2′′, H-6′′), 6.90 (2 H, d, *J* = 8.8, H-3′, H-5′), 5.31 (1 H, q, *J* = 7.04, CH*), 3.88 – 4.08 (2 H, m, $COOCH_2$), 3.68 (2 H, t, *J* = 6.7, OCH_2), 2,18 (2 H, t, *J* = 7,0, CH_2SH), 1.73–1.96 (4 H, m, C**H**$_2CH_2O$, C**H**$_2CH_2SH$), 1.42–1.55 (5 H, m, C**H**$_2CH_2OOC$, C**H**$_3CH$*), 1.08–1.35 (16 H, m, 8 × CH_2), 0.75 (t, 3 H, *J* = 6.7, CH_2C**H**$_3$).

(S)-1-(decyloxy)-1-oxopropan-2-yl 4'-((4'''-((10-bromodecyl)oxy)-[1'',1'''-biphenyl]-4-carbonyl)oxy)-3'-chloro-[1,1'-biphenyl]-4-carboxylate (9)

Same as in the case of bromo-ester **7**: the acid **3** (12.0 g, 27.69 mmol) was condensed with phenol **8** (12.80 g, 27.77 mmol) using DCC (6.30 g, 30.23 mmol) and DMAP (1.0 g, 7.94 mmol) gave 24.60 g (79 %) of **9** after column chromatography on silica using dichloromethane-acetone (98:2) as eluent. ^{1}H NMR ($CDCl_3$): 8.29 (2 H, d, *J* = 8.8, H-3′′, H-5′′), 8.18 (2 H, d, *J* = 8.2, H-3, H-5), 7.75 (1 H, d, *J* = 2.3,

H-5′), 7.72 (2 H, d, J = 8.8, H-2′′, H-6′′), 7.67 (2 H, d, J = 8.2, H-2′′′, H-6′′′), 7.55–7.63 (3 H, m, H-2, H-6, H-6′), 7.41 (1 H, d, J = 8.2, H-5′), 7.01 (2 H, d, J = 8.8, H-3′′′, H-5′′′), 5.36 (2 H, q, J = 7.0, C*H), 4.10–4.29 (2 H, m, C*HCOOC**H**$_2$), 4.02 (3 H, t, J = 6.7, CH_2O), 3.41 (2H, t, J = 6.5, CH_2Br), 1.75–1.93 (2 H, m, C**H**$_2$$CH_2O$), 1.57–1.73 (5 H, m, $COOCH_2$C**H**$_2$, CH*C**H**$_3$), 1.11–1.55 (26 H, m, 13 × CH_2), 0.88 (3 H, t, J = 6.7, CH_2C**H**$_3$).

(S)-1-(decyloxy)-1-oxopropan-2-yl 3'-chloro-4'-((4'''-((10-sulfanyldecyl)oxy)-[1'',1'''-biphenyl]-4-carbonyl)oxy)-[1,1'-biphenyl]-4-carboxylate (Thiol C)

Thiol C was synthesized using the procedure described for **Thiol B**. Starting from bromo-ester **9** (19.0 g, 21.68 mmol), which was converted to Bunte salt by reaction with aqueous sodium thiosulphate (32.0 g, 202.39 mmol) in ethanol-dioxane mixture and subsequent decomposition by diluted HCl in water-THF mixture under inert atmosphere yielded 5.33 g (30 %) of **Thiol C**. ^{1}H NMR (C_6D_6): 8.38 (2 H, d, J = 8.8, H-3′′, H-5′′), 8.18 (2 H, d, J = 8.2, H-3, H-5), 7.77 (1 H, d, J = 2.3, H-5′), 7.73 (2 H, d, J = 8.8, H-2′′, H-6′′), 7.69 (2 H, d, J = 8.2, H-2′′′, H-6′′′), 7.1–7.38 (3 H, m, H-2, H-6, H-6′), 7.00 (1 H, d, J = 8.2, H-5′), 6.90 (2 H, d, J = 8.8, H-3′′′, H-5′′′), 5.38 (2 H, q, J = 7.0, C*H), 3.80–4.11 (2 H, m, C*HCOOC**H**$_2$), 4.01 (3 H, t, J = 6.7, CH_2O), 2.18 (2H, t, *m*, CH_2SH), 1.74–1.90 (2 H, m, C**H**$_2$$CH_2O$), 1.55–1.71 (5 H, m, $COOCH_2$C**H**$_2$, CH*C**H**$_3$), 1.10–1.51 (26 H, m, 13 × CH_2), 0.87 (3 H, t, J = 6.7, CH_2C**H**$_3$).

2. Preparation of Silver Nanoparticles

2.1. Synthesis of Silver Nanoparticle System NP1

To the solution of 1.02 g $AgNO_3$ in 1.0 l of water/ethanol (1:1) mixture preheated to 60 °C there was added slowly dropwise during several hours under intensive stirring 6 ml of tannine solution (1.0 g/100 mL H_2O), 6 ml of sodium carbonate solution (1.0 g/100 mL H_2O) and solution of 1 g mesogenic **Thiol A** in 50 mL of benzene. The dark brown benzene layer was separated and water layer extracted by hot benzene again. Combined benzene solutions were filtered and after partial evaporation, the nanoparticles were precipitated by addition of ethanol. The supernatant was removed and the solid phase was dissolved in tetrahydrofurane, precipitated by ethanol again and centrifuged. The procedure was repeated until the signals of thiol and residual disulphide disappeared.

The signals in C_6D_6:

- CH_2-SH	^{1}H: 2.2 ppm, quartet, J = 8.7 Hz	^{13}C: 24.7 ppm
- CH_2-S-Ag	^{1}H: 2.6 ppm, triplet, J = 7.29 Hz	^{13}C: 28.9 ppm

2.2. Preparation of NP2

Thiol B (2.0 g, 3.1 mmol) was dissolved in ethanol (500 mL) at 60 °C under argon atmosphere, and a solution of $AgNO_3$ (0.2 g, 1.2 mmol) in demi-water (1.0 l) was added. The resulting mixture was treated with solution of sodium borohydride (1.5 g, 4.0 mmol) in demi-water (100 mL) over 1 h. The resulting dark suspension was filtered, and the solid dissolved in chloroform and filtered again. Obtained filtrate was evaporated under reduced pressure, and the residue further purified by precipitation from boiling toluene by the addition of acetonitrile. Additional purification included triple centrifugation of suspension in acetonitrile. Yield=560 mg.

2.3. Synthesis of Silver Nanoparticle Systems NP3 and NP4

Three solutions were prepared. Solution A—5.0 g silver nitrate was dissolved in 200 mL of distilled water and keep in dark. Solution B—9.0 g potassium hydroxide was dissolved in 200 mL of water. Solution C—8.0 g succrose was boiled for 30 min in mixture of 80 mL distilled water, and 10 mL of ethanol acidified by 0.35 mL nitric acid (65 %).

NP3: To the solution A (32 mL), concentrated ammonium hydroxide (25%) was added drop wise under vigorous stirring until the precipitation, which occurred initially, was completely dissolved. Then, 8 mL of solution B was added and ammonium hydroxide added dropwise again until

generated precipitate is dissolved and the solution was clear. This process was repeated once more. The mixture was kept in refrigerator (0 °C) for 30 min, 2 mL of solution C was added, and the resulting mixture was poured into 500 mL of distilled water. To this solution (which contains 0.5 g of silver) 5 g of Thiol C in 200 mL of toluene was added under stirring at 25 °C within 1 h. The resulting mixture was stirred for 3 h. The dark brown upper layer was separated after the addition of small amount of ethanol, filtered over paper filter, and evaporated under reduced pressure to the volume 5–10 mL. The nanoparticles were precipitated by the excess of ethanol and centrifuged. The solid phase was dissolved in hot tetrahydrofurane and precipitated by ethanol again. This process was repeated several times until all free thiol molecules were removed, which was confirmed by nuclear magnetic resonance (NMR) spectrum. Yield 520 mg, as black powder.

The nanoparticle system NP4 was prepared by the same procedure and quantities as the nanoparticles NP3. The only modification was that the mixture of solutions A + B + C was poured into distilled water (300 mL) with ice (200 g) to perform the reaction at a lower temperature. The total reaction time was same (4 h). The yield was 450 mg NP4 as black powder.

3. Example of NMR Spectra of Free Thiol and Nanoparticle System

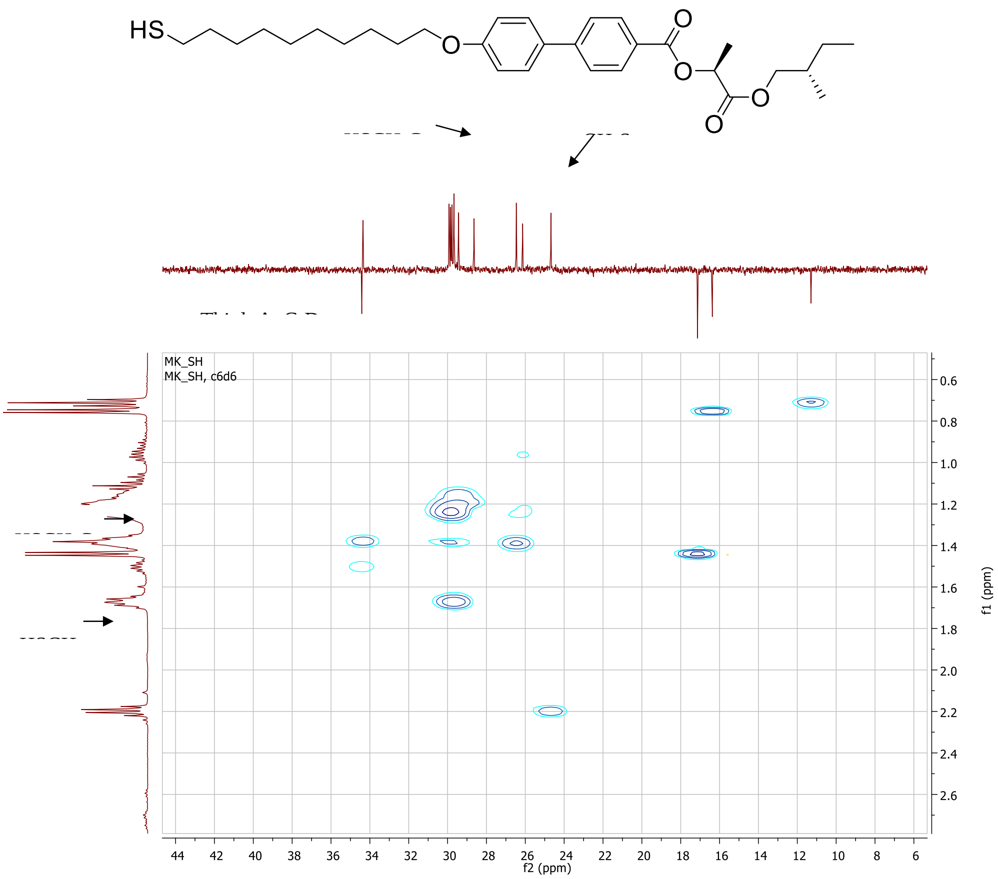

Figure S1. aliphatic region of heteronuclear single quantum coherence (HSQC) spectrum of Thiol A in C_6D_6.

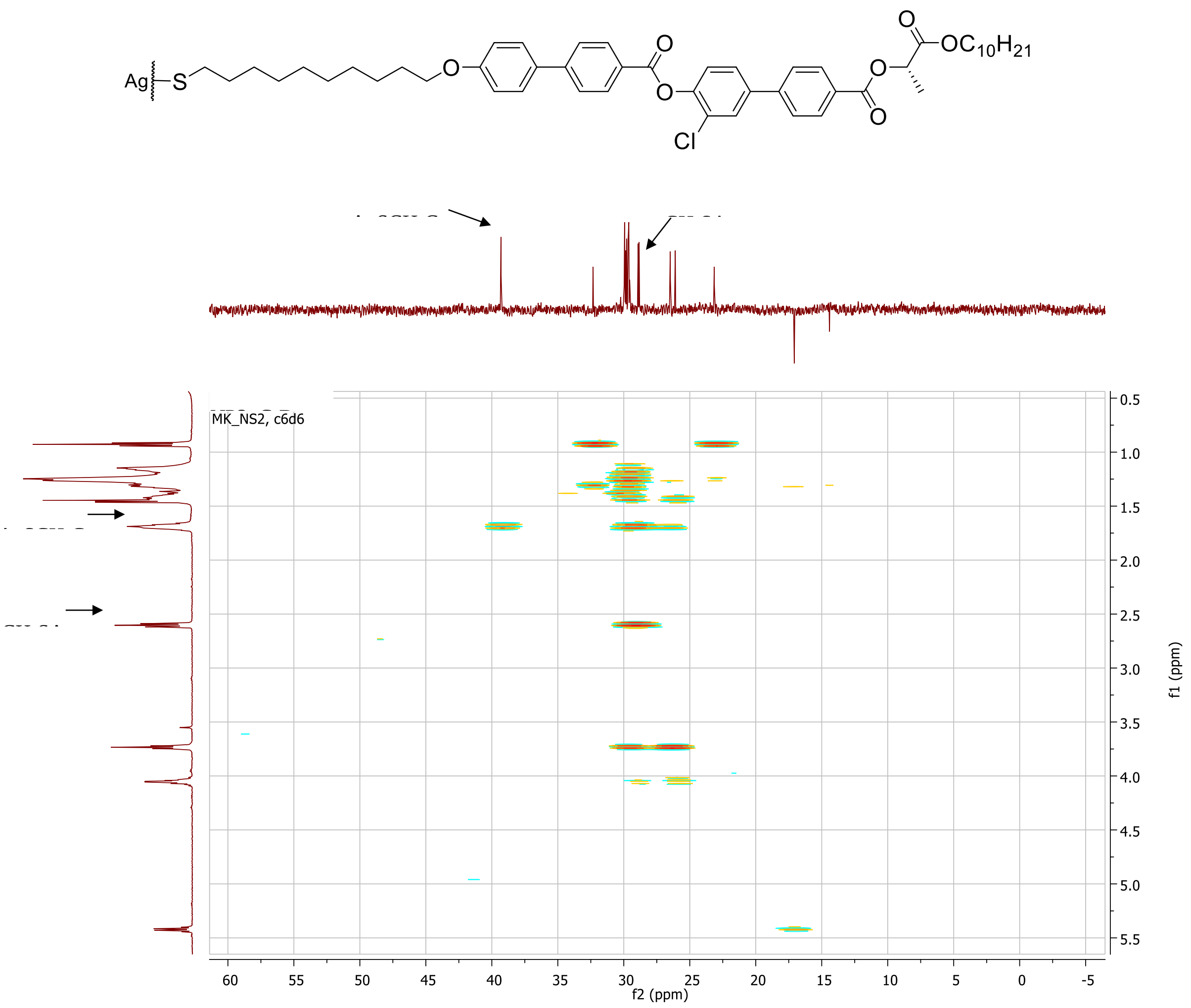

Figure S2. Aliphatic region of heteronuclear multipole bond correlation (HMBC) spectrum of NP3 in C_6D_6.

4. Results

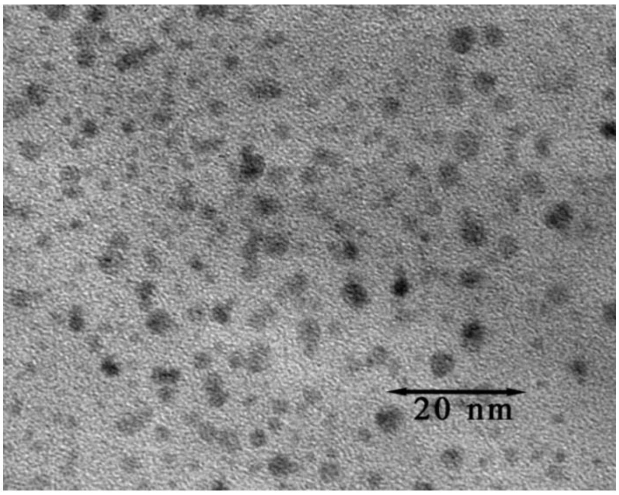

Figure S3. TEM picture of NP4.

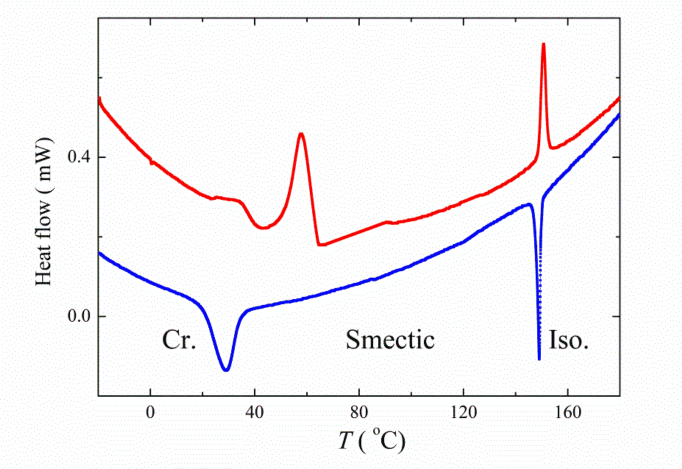

Figure S4. Thermograph for NP2 shows the second heating (red colour) and the second cooling runs.

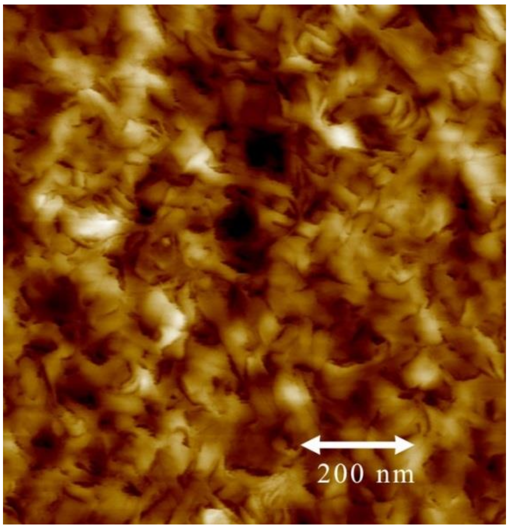

Figure S5. Atomic force microscopy (AFM) image on film created from NP4 at the room temperature, freely evaporated from a solution in toluene.

Table S1. The melting points (m.p.) taken on the second heating, the phase transition temperature smectic-isotropic, T_{iso}, and the crystallization temperature, T_{cr}, on the second cooling in °C and corresponding enthalpy changes, ΔH, in J/g, are in brackets at the corresponding temperature. The symbol * shows that the crystallization is not fully completed on the second cooling, and an additional crystallization peak appears on subsequent heating.

	**m.p. [H]**	**T_{iso} on heating [H]**	**T_{iso} on cooling [H]**	**T_{cr} [H]**
NP2	53 [+13.8]	150 [+5.07]	150 [−4.10]	34 [−10.2]
NP4	58 [+25.5]	180 [+0.82]	180 [−0.80]	40 [−1.52]*

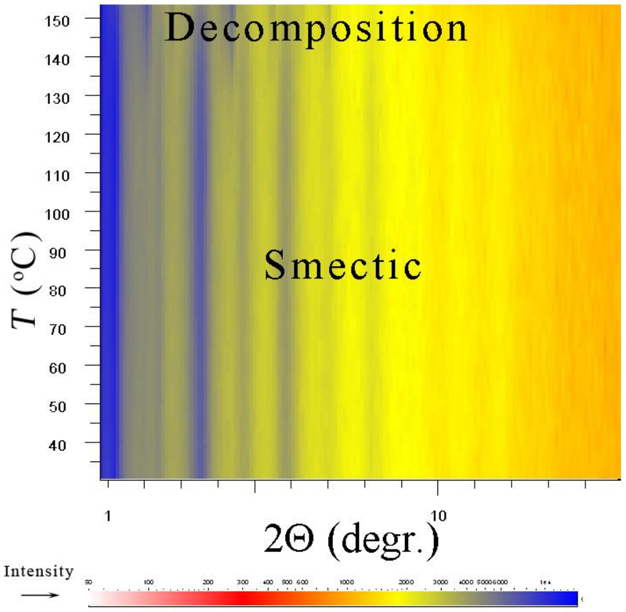

Figure S6. Dependences of the X-ray intensity (corresponding colours are below the graph) for compound NP2. The figure shows a distinct smectic-like mesophase on heating and decomposition at higher temperatures above 140ºC.

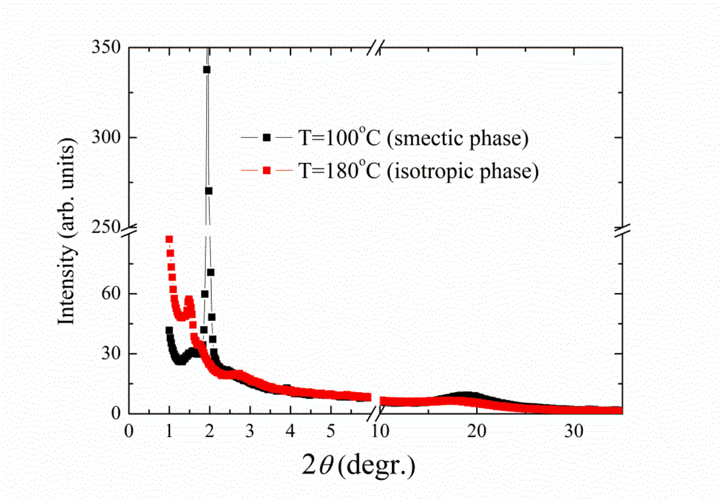

Figure S7. Intensity profile with respect to the scattering angle for NP2 in the smectic phase at T = 100 °C and in the isotropic phase at T = 180 °C for comparison.

5. References

1. V. Novotná, M. Kašpar, V. Hamplová, M. Glogarová, P. Bílková, V. Domenici, D. Pociecha, *Liq. Cryst.*, 2008, **35**, 287–298.
2. M. Kašpar, V. Hamplová, V. Novotná, M. Glogarová, D. Pociecha, P. Vaněk. New series of ferroelectric liquid crystals with two or three chiral centers exhibiting antiferroelectric and hexatic phases. *Liq. Cryst.* 2001, 28, 1203–1209.
3. M. Kašpar, V. Hamplová, S. A. Pakhomov, A. M. Bubnov, F. Guittard, H. Sverenyak, I. Stibor, P. Vaněk, M. Glogarová, *Liq. Cryst.*, 1998, **24**, 599–605
4. V. Novotná, V. Hamplová, N. Podoliak, M. Kašpar, M. Glogarová, D. Pociecha, E. Gorecka. *J. Mater. Chem.*, 2011, **21**, 14807–14814.
